# Trastuzumab-resistant breast cancer cells-derived tumor xenograft models exhibit distinct sensitivity to lapatinib treatment in vivo

**DOI:** 10.1186/s12575-023-00212-3

**Published:** 2023-06-27

**Authors:** Hao Liu, Sanbao Ruan, Margaret E. Larsen, Congcong Tan, Bolin Liu, Hui Lyu

**Affiliations:** 1 Departments of Interdisciplinary Oncology and Genetics, Stanley S. Scott Cancer Center, School of Medicine, Louisiana State University (LSU) Health Sciences Center, 1700 Tulane Ave., New Orleans, LA 70112 USA; 2 Affiliated Cancer Hospital & Institute of Guangzhou Medical University, Guangzhou, Guangdong China

**Keywords:** Therapeutic resistance, HER2-targeted therapy, In vivo tumor models, Breast cancer

## Abstract

**Background:**

Resistance to HER2-targeted therapies, including the monoclonal antibody trastuzumab and tyrosine kinase inhibitor lapatinib, frequently occurs and currently represents a significant clinical challenge in the management of HER2-positive breast cancer. We previously showed that the trastuzumab-resistant SKBR3-pool2 and BT474-HR20 sublines were refractory to lapatinib in vitro as compared to the parental SKBR3 and BT474 cells, respectively. The in vivo efficacy of lapatinib against trastuzumab-resistant breast cancer remained unclear.

**Results:**

In tumor xenograft models, both SKBR3-pool2- and BT474-HR20-derived tumors retained their resistance phenotype to trastuzumab; however, those tumors responded differently to the treatment with lapatinib. While lapatinib markedly suppressed growth of SKBR3-pool2-derived tumors, it slightly attenuated BT474-HR20 tumor growth. Immunohistochemistry analyses revealed that lapatinib neither affected the expression of HER3, nor altered the levels of phosphorylated HER3 and FOXO3a in vivo. Interestingly, lapatinib treatment significantly increased the levels of phosphorylated Akt and upregulated the expression of insulin receptor substrate-1 (IRS1) in the tumors-derived from BT474-HR20, but not SKBR3-pool2 cells.

**Conclusions:**

Our data indicated that SKBR3-pool2-derived tumors were highly sensitive to lapatinib treatment, whereas BT474-HR20 tumors exhibited resistance to lapatinib. It seemed that the inefficacy of lapatinib against BT474-HR20 tumors in vivo was attributed to lapatinib-induced upregulation of IRS1 and activation of Akt. Thus, the tumor xenograft models-derived from SKBR3-pool2 and BT474-HR20 cells serve as an excellent in vivo system to test the efficacy of other HER2-targeted therapies and novel agents to overcome trastuzumab resistance against HER2-positive breast cancer.

## Introduction

Amplification/overexpression of *HER2* (also known as e*rbB2/neu*) is significantly associated with poor prognosis in breast cancer (BC) patients [1, 2]. HER2-targeted therapies, including trastuzumab (Herceptin) and lapatinib (Tykerb) are commonly used in the clinic and have dramatically improved the survival of BC patients with HER2-overexpressing (HER2-positive) tumors [3, 4]. It is well-known that trastuzumab is a humanized anti-HER2 monoclonal antibody (mAb) binding to the extracellular domain of HER2, blocks its signaling. Lapatinib, as a small molecule tyrosine kinase inhibitor (TKI), mainly inhibits the kinase activity of HER2 via targeting its intracellular domain. Although trastuzumab and lapatinib are effective for metastatic BC patients with HER2-positive tumors [5–8], both primary (de novo) and acquired resistance to the HER2-targeted therapies frequently occur and currently represent a significant clinical challenge for successful treatment of HER2-positive BC [9]. To date, we lack validated biomarkers to predict the response of HER2-positive BC to these therapies [4, 10]. There is an unmet need to identify novel approaches/agents to overcome the resistance with an aim to improve the survival of HER2-positive BC patients, especially those with advanced/metastatic diseases.

Several mechanisms of resistance to HER2-targeted therapy have been proposed in the treatment of HER2-positive BC [3, 9]. Among them, activation of compensatory signaling pathways-triggered by alternative receptor tyrosine kinases (RTKs), including HER3 and the insulin-like growth factor-1 (IGF-1R), plays an important role in attenuating the efficacy of an HER2-targeted therapy [11–13]. Whether HER2-positive BC cells utilize similar or different mechanisms to create resistance to trastuzumab and lapatinib remains an open question in our understanding of the molecular basis of HER2-targeted therapy resistance. Some studies have shown that activation of the signaling pathways initiated by other HER family members, such as EGFR and HER3, or non-HER receptors, including AXL, limits the response of HER2-positive BC cells to both trastuzumab and lapatinib [14–18]. Other reports suggest that the two agents may not share common mechanisms of resistance. While the PI-3K/Akt signaling pathway has been implicated as a major determinant of trastuzumab resistance [19], its role in lapatinib resistance remains elusive. Loss of PTEN-triggered PI-3K/Akt signaling has been shown to result in lapatinib resistance, which can be overcome by NVP-BEZ235, a dual inhibitor of PI-3K/mTOR [20]. Yet, other studies indicate that activation of the PI-3K/Akt signaling confers resistance to trastuzumab, but not lapatinib [21, 22], and lapatinib potently suppresses tumor growth of HER2-positive BC in a PTEN-independent manner [23].

The PI-3K/Akt signaling is one of the major downstream signaling pathways-initiated by HER3 and IGF-1R, and critically contributes to trastuzumab resistance [24–26]. We investigated the relationship of HER3 and IGF-1R in HER2-positive BC cells with acquired resistance to trastuzumab. Our data showed that both HER3 and IGF-1R directly interacted with HER2 in the trastuzumab-resistant BC sublines SKBR3-pool2 and BT474-HR20, which were derived from the parental BC cell lines SKBR3 and BT474, respectively [27]. In the resistant cells, HER2, HER3, and IGF-1R formed a heterotrimeric complex, which was responsible for enhanced activation of multiple downstream pathways, including the PI-3K/Akt signaling and Src kinase [27]. We next wondered whether the trastuzumab-resistant sublines would retain their resistant phenotypes to lapatinib. Our data showed that SKBR3-pool2 and BT474-HR20 cells as compared to their sensitive counterparts were relatively refractory to lapatinib treatment under our cell culture conditions [28]. Further examination revealed that HER3 and IGF-1R initiated distinct signaling pathways altering the responses of SKBR3-pool2 and BT474-HR20 cells to lapatinib in vitro [28]. In the current studies, we took advantage of the tumor xenograft models established with SKBR3-pool2 or BT474-HR20 cells in nude mice to determine the in vivo antitumor activity of lapatinib against trastuzumab-resistant BC.

## Results

### Trastuzumab-resistant breast cancer sublines maintain their resistance phenotypes in vivo

**Fig. 1:**
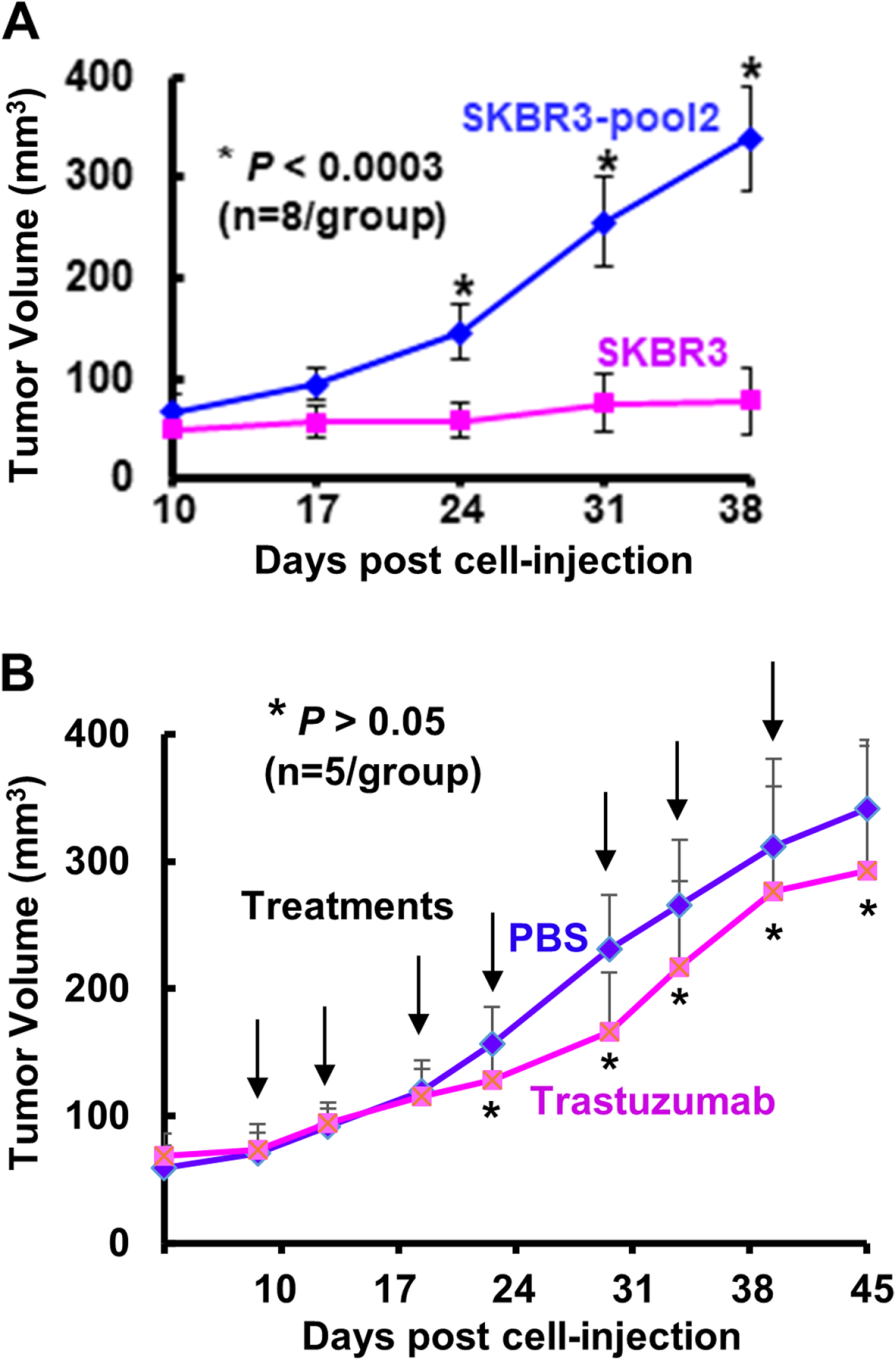
SKBR3-pool2 cells as compared to the parental SBKR3 cells grew tumors in nude mice with a significantly faster rate and retained trastuzumab resistance phenotype in vivo. **A** SKBR3 or SKBR3-pool2 cells (5 × 10^6^) were injected s.c into the flanks of 5-week-old female nude mice (*n* = 8/group). Mice were observed three times and tumor formation was measured twice a week. Tumor volume was calculated and expressed as cubic millimeters (mean ± SD). **B** When tumor volumes reached ~ 80mm3, the mice bearing tumors-derived from SKBR3-pool2 cells were treated with either control (PBS) or trastuzumab (20 mg/kg) twice a week for seven times (*n* = 5/group). Tumor volume was expressed as cubic millimeters (mean ± SD). Treatment with trastuzumab had no significant effect on SKBR3-pool2 tumor growth at all-time points examined. Statistical analyses were performed with two-sided student’s t tests

We previously reported that trastuzumab-resistant BT474-HR20 cells formed tumors in nude mice with a much shorter latency than parental BT474 cells [29]. Moreover, BT474-HR20-derived tumors were significantly less sensitive to trastuzumab treatment [29]. To determine if all trastuzumab-resistant BC cells would display aggressive characteristics and retain resistant phenotypes to trastuzumab in vivo, we performed similar experiments with the tumor xenograft models established from the trastuzumab-resistant SKBR3-pool2 cells in comparison with that established from the parental SKBR3 cells. We found that the tumors derived from SKBR3-pool2 cells grew significantly faster than that derived from SKBR3 cells (Fig. 1A). Treatment with trastuzumab had no significant effect on the growth of SKBR3-pool2 tumors at all-time points examined (Fig. 1B). The experiment was terminated at day 45 post cell injection. Our data indicated that although BT474-HR20 and SKBR3-pool2 were originally obtained via in vitro cell culture studies, they both maintained their trastuzumab resistance phenotypes in vivo. The aggressiveness of BT474-HR20 and SKBR3-pool2 cells to form tumors in nude mice enabled them to serve as excellent in vivo models to examine the efficacy of other therapeutic agents against trastuzumab-resistant BC.

### SKBR3-pool2 and BT474-HR20-derived tumors show distinct sensitivity to lapatinib in in vivo xenograft models

**Fig. 2:**
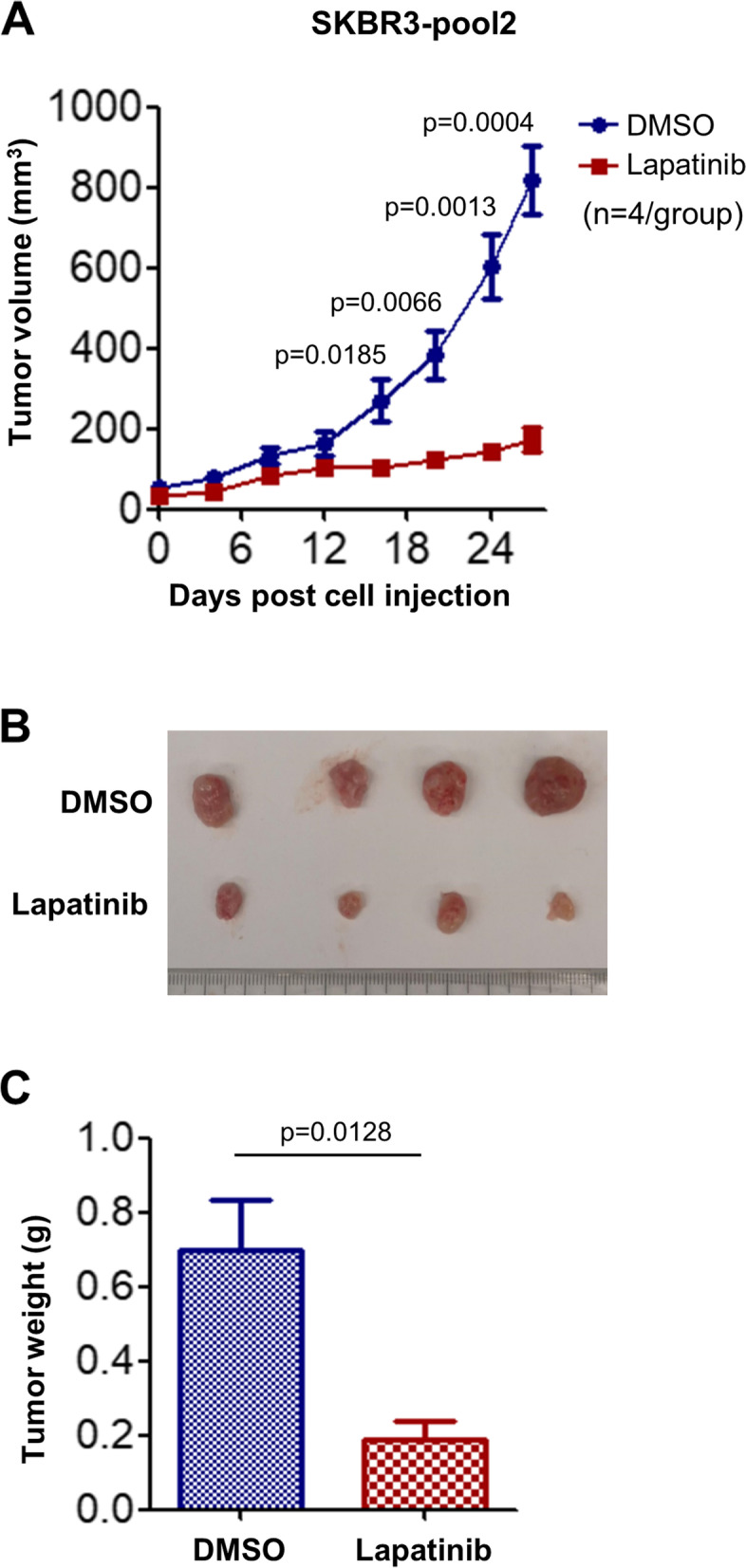
Lapatinib exhibited potent inhibitory effects on SKBR3-pool2 tumor growth in vivo. SKBR3-pool2 cells (8 × 10^6^) were injected s.c into the flanks of 5-week-old female nude mice. When tumor volumes reached ~ 80 mm^3^, mice were treated with vehicle control (DMSO) or lapatinib (80 mg/kg, i.p.) every other day (*n* = 4/group). **A** Tumor growth curves were plotted using average tumor volume within each group at the indicated time points. Bars, SD. **B **&** C** at the end of the experiment, all mice were sacrificed. The tumors were dissected, imaged as indicated (**B**) and measured for weight (**C**). Two-sided student’s t tests were used for statistical analyses

**Fig. 3:**
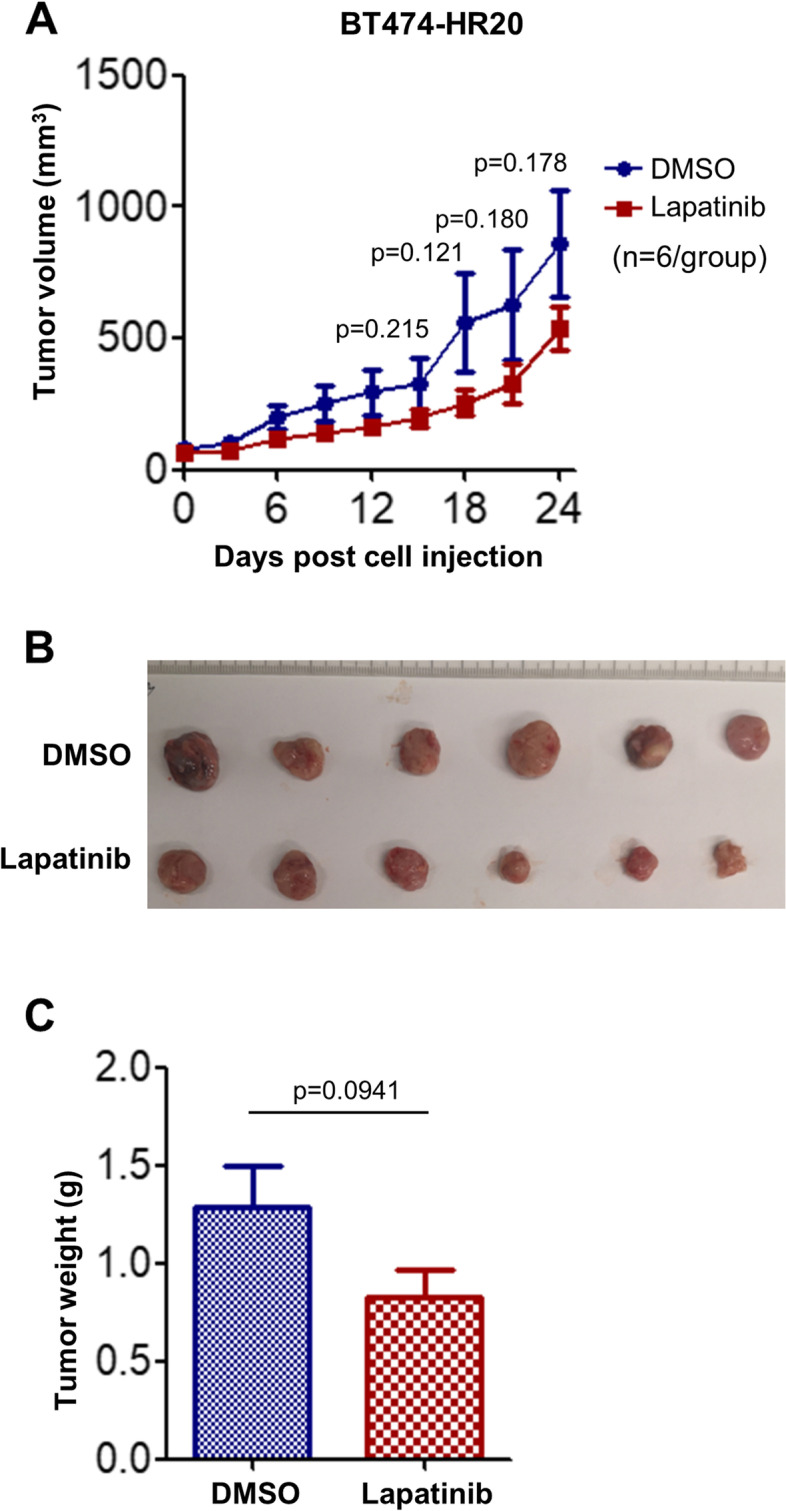
Lapatinib slightly attenuated the growth of BT474-HR20-derived tumors in xenograft models. BT474-HR20 cells (8 × 10^6^) were injected s.c into the flanks of 5-week-old female nude mice. When tumor volumes reached ~ 80 mm^3^, mice were treated with vehicle control (DMSO) or lapatinib (80 mg/kg, i.p.) every other day (*n* = 6/group). **A** Tumor growth curves were plotted using average tumor volume within each group at the indicated time points. Bars, SD. **B **&** C** at the end of the experiment, all mice were sacrificed. The tumors were dissected, imaged as indicated (**B**) and measured for weight (**C**). Two-sided student’s t tests were used for statistical analyses

To test whether lapatinib would exert its antitumor effect on trastuzumab-resistant BC in vivo, we again used SKBR3-pool2 or BT474-HR20 cells to establish tumor xenograft models in nude mice. The tumor-bearing mice were treated with either vehicle control (DMSO) or lapatinib via intraperitoneal injection. At the end of the experiments, all tumors were photographed and weighted. Treatment with lapatinib profoundly suppressed SKBR3-pool2 tumor growth (Fig. 2A). The tumors obtained from lapatinib-treated mice were much smaller and significantly lighter than that obtained from controls (Fig. 2B & C). In contrast, the tumors-derived from BT474-HR20 cells were less sensitive to lapatinib treatment. Lapatinib slightly, but not significantly inhibited BT474-HR20 tumor growth (Fig. 3A). The size of the tumors obtained from lapatinib-treated mice was similar to those obtained from controls (Fig. 3B). There was no significant difference in the tumor weight between the two groups (Fig. 3C). Collectively, these data demonstrated that both SKBR3-pool2 and BT474-HR20 maintained their trastuzumab resistance phenotypes in vivo ( Fig. 1 and [29]), and they also were refractory to lapatinib treatment in in vitro cell culture experiments [28]. However, lapatinib exhibited different antitumor activity in xenograft models established from SKBR3-pool2 or BT474-HR20 cells. While lapatinib markedly inhibited SKBR3-pool2 tumor growth, it had little effect on BT474-HR20 tumor growth. Our studies suggest that some, but not all trastuzumab-resistant BCs respond well to lapatinib in in vivo tumor xenograft models.

### Lapatinib differentially regulates the levels of phosphorylated Akt (p-Akt) and IRS1 expression in SKBR3-pool2- and BT474-HR20-derived tumors

**Fig. 4:**
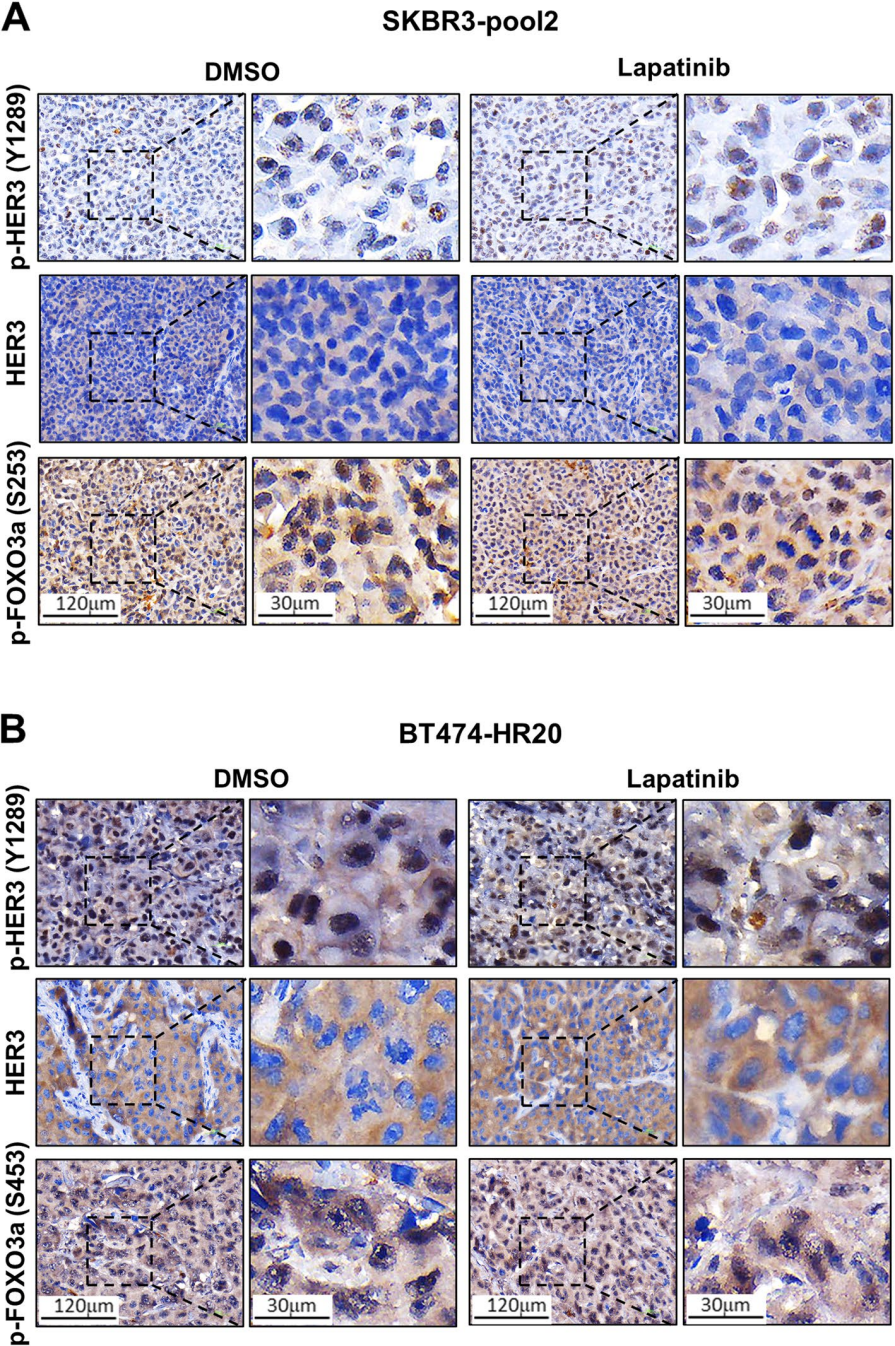
No significant changes of p-HER3, HER3, and p-FOXO3a were observed between DMSO- and lapatinib-treated tumors derived from SKBR3-pool2 or BT474-HR20 cells. The tumors derived from either SKBR3-pool2 (**A**) or BT474-HR20 (**B**) cells were formalin-fixed and paraffin-embedded (FFPE) and sectioned into five-micron-thick slides. The FFPE slides were analyzed with IHC staining assays for p-HER3 (Y1289), HER3, or p-FOXO3a (Ser253) following the procedures described in the materials and methods. Two individuals independently evaluated the IHC staining. The levels of p-HER3, HER3, and p-FOXO3a showed no apparent differences between control and lapatinib-treated groups

**Fig. 5:**
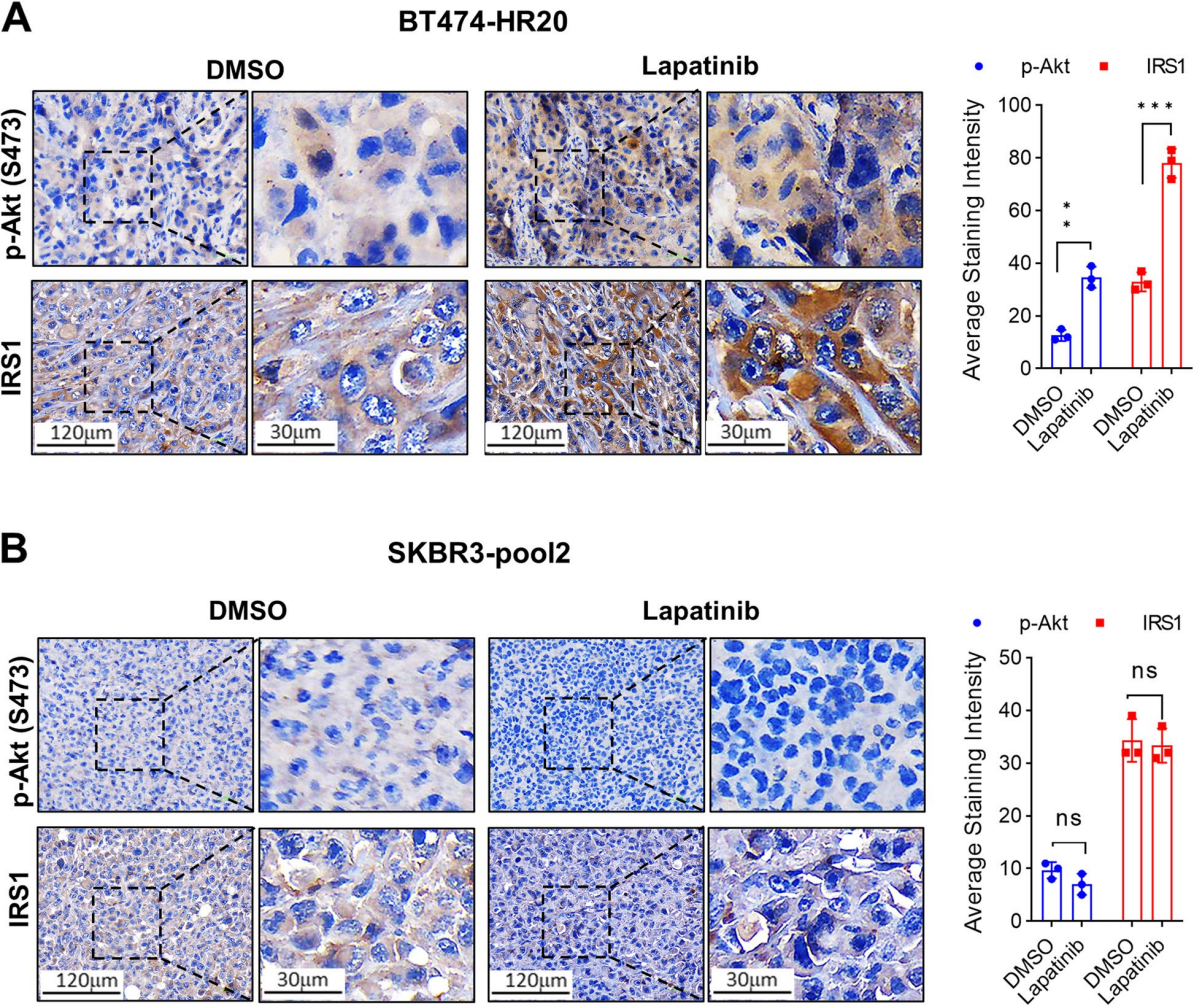
Lapatinib treatment markedly increased p-Akt levels and enhanced IRS1 expression in the tumors-derived from BT474-HR20, but not SKBR3-pool2 cells. The FFPE slides of BT474-HR20 (**A**) or SKBR3-pool2 (**B**) tumors were analyzed with IHC staining assays for p-Akt (Ser473) or IRS1. The IHC staining was evaluated by two individuals independently and quantified with ImageJ and ImageJ plugin IHC profiler. The average staining intensity of three random field of each slide was plotted and shown as bar graphs on the right. The data were statistically analyzed with two-sided student’s t tests. *Bars*, SD. ns, not significant. ** *p* < 0.01, *** *p* < 0.005

We observed an enhanced activation of multiple signaling pathways, including PI-3K/Akt and Src kinase in SKBR3-pool2 and BT474-HR20 cells because of the trimeric complex formed by HER2, HER3, and IGF-1R [27]. Specific inhibition of either Akt or Src reversed the resistant phenotypes of both SKBR3-pool2 and BT474-HR20 cells [27]. This was consistent with other reports which indicated that PI-3K/Akt signaling and Src kinase played a pivotal role in the development of trastuzumab resistance [19, 30, 31]. Our studies also showed that HER3 induced activation of both PI-3K/Akt signaling and Src, whereas IGF-1R mainly activated Src kinase in SKBR3-pool2 and BT474-HR20 cells [28]. Further defining the underlying mechanisms of HER3-/IGF-1R-initiated signaling in trastuzumab resistance, we recently discovered elevated levels of phosphorylated FOXO3a (p-FOXO3a) in the trastuzumab-resistant cells due to transcriptional suppression of PPP3CB, which is a subunit of the serine/threonine-protein phosphatase 2B (PP2B) [32]. The increased p-FOXO3a disrupted a negative feedback inhibition loop formed by FOXO3a and several *IGF2*-/*IRS1*-targeting miRNAs (miR-193a-5p, miR-128-3p, and miR-30a-5p). This, thereby, increased the expression of both IGF2 and IRS1 [32]. Thus, we hypothesized that the distinct antitumor activity of lapatinib against SKBR3-pool2 and BT474-HR20 in vivo might be attributed to its differential effects on the aberrant activation of HER3 (phosphorylation) and/or the IGF2/IGF-1R/IRS1 signaling in the resistant tumors. To this end, we took advantage of the SKBR3-pool2 and BT474-HR20 tumors obtained from the animal experiments and performed immunohistochemistry (IHC) assays on HER3, p-HER3, p-FOXO3a, p-Akt, and IRS1. For both SKBR3-pool2 and BT474-HR20 tumors, we found no apparent difference in the levels of HER3, p-HER3, and p-FOXO3a between lapatinib-treated mice and controls (Fig. 4A & B), i.e. lapatinib treatment had little effect on HER3 protein expression and activation in vivo. In contrast, the levels of p-Akt and IRS1 were markedly increased in the BT474-HR20 tumors obtained from lapatinib-treated mice as compared to that from controls (Fig. 5A left). Since lapatinib treatment generally had no effect on Akt expression, we focused our studies on p-Akt. Quantification analyses of both p-Akt and IRS1 were significantly different between the two groups (Fig. 5A right). Taken together, our data suggest that lapatinib’s inefficacy against BT474-HR20 tumors in xenograft models is likely due to its capability to induce Akt activation (phosphorylation) and/or enhance IRS1 expression in vivo.

## Discussion

Lapatinib is an orally bioavailable reversible TKI with dual targeting function against both EGFR and HER2 [33]. Its combination with capecitabine is indicated to treat patients with advanced/metastatic HER2-positive BC whose diseases have progressed after prior therapy with trastuzumab plus chemotherapy [33, 34]. Despite lapatinib’s success in HER2-positive BC patients being observed, both primary (de novo) and acquired resistances to lapatinib frequently occur in the clinical setting [35, 36]. Among those potential mechanisms of lapatinib resistance, reactivation of the downstream signaling pathways, especially the PI-3K/Akt signaling has been shown to play an important role in the development of resistance to lapatinib [34, 37]. In the current studies using tumor xenograft models established with the trastuzumab-resistant BC sublines, we observed a marked increase of p-Akt in BT474-HR20-derived tumors (Fig. 5A left), which were refractory to lapatinib treatment (Fig. 3A). In contrast, lapatinib exerted profound suppression on SKBR3-pool2 tumor growth (Fig. 2A), and there was no increase in p-Akt levels in the SKBR3-pool2 tumors of lapatinib-treated mice as compared to controls (Fig. 5B left). Collectively, our data suggest that enhanced activation of Akt (evidenced by increased p-Akt) likely contributes to the inefficacy of lapatinib against BT474-HR20 tumor growth in vivo.

The underlying mechanisms resulting in increased p-Akt in the BT474-HR20 tumors after lapatinib treatment remain unclear. Accumulating evidence indicates that compensatory induction of HER3 potently activates the PI-3 K/Akt signaling pathway, thereby leading to resistance to a wide variety of therapeutic agents [12, 38], including lapatinib [15]. However, we did not detect apparent alterations in either HER3 protein levels or its phosphorylation (p-HER3) in both BT474-HR20 and SKBR3-pool2 tumors upon lapatinib treatment (Fig. 4). This suggest that reactivation of Akt in lapatinib-treated BT474-HR20 tumors is likely, via a mechanism independent of HER3 upregulation and activation. We previously showed that increased p-FOXO3a and elevated expression of IGF2 and IRS1 were critical for the development of trastuzumab resistance in HER2-positive BC. This occurred via disruption of a negative feedback inhibition loop of FOXO3a and several *IGF2*-/*IRS1*-targeting miRNAs [32]. Here, our IHC analyses found little change in p-FOXO3a levels between control and lapatinib-treated BT474-HR20 and SKBR3-pool2 tumors (Fig. 4), whereas the expression of IRS1 was significantly increased upon lapatinib treatment only in BT474-HR20 tumors (Fig. 5). Interestingly, recent investigation showed that the expression of IRS4, a closely related member of the IRS family (consisting of IRS1, IRS2, and IRS4) activated the PI-3K/Akt signaling without significant activation of the upstream RTKs, and subsequently caused resistance to HER2-targeted therapy [39, 40]. Nonetheless, whether the elevated expression of IRS1 is responsible for the increased p-Akt levels in lapatinib-treated BT474-HR20 tumors remains unknown. It is also unclear if lapatinib treatment may upregulate IRS4 in the BT474-HR20 tumors. Alternatively, the increased p-Akt may be due to activation of the non-receptor tyrosine kinase Src [41], which functions as a key mechanism of trastuzumab resistance and indicates poor prognosis in HER2-positive/ER-negative BC [42]. Src activation has been shown to cause resistance to lapatinib in HER2-positive BC cells [43, 44]. Our studies of SKBR3-pool2 and BT474-HR20 cells revealed that IGF-1R-initiated Src activation did not influence the efficacy of lapatinib under in vitro cell culture conditions [28]. Currently, we are performing IHC analysis of Src and p-Src to examine if lapatinib treatment in vivo may alter Src expression and/or activation in the tumors derived from BT474-HR20 or SKBR3-pool2 cells.

In 2010, lapatinib was approved for use with an aromatase inhibitor (letrozole) in the treatment of post-menopausal women with metastatic BC co-expressing hormonal receptors (estrogen receptor (ER) and progesterone receptor (PR)) and HER2 [34, 36]. ER expression and/or activation have been shown to contribute to the development of different mechanisms of resistance to trastuzumab and lapatinib [45, 46]. BT474 cells are also ER-positive, a typical luminal B subtype of BC cells, whereas SKBR3 cells do not express ER/PR. Whether the expression of ER may play a role in the resistant phenotype of BT474-HR20 tumors to lapatinib treatment in vivo remains to be determined. It would be interesting and clinically relevant to test whether the combinations of lapatinib and letrozole may synergistically inhibit BT474-HR20 tumor growth in our xenograft models.

In summary, we demonstrate that trastuzumab-resistant BCs exhibit distinct sensitivity to the treatment of lapatinib in tumor xenograft models. Enhanced activation of Akt (evidenced by increased p-Akt) and the elevated expression of IRS1 seem to be associated with the resistance of BT474-HR20 tumors to lapatinib treatment in vivo. Our data support that SKBR3-pool2- and BT474-HR20-derived tumor xenograft models serve as an excellent in vivo system to test the antitumor activity of other HER2-targeted therapies, including lapatinib against trastuzumab-resistant BC. The model system may also be used to determine the potential of novel therapeutic agents to overcome trastuzumab resistance in HER2-positive BC.

## Materials and methods

### Reagents and antibodies

Trastuzumab (Herceptin) was purchased from Roche (Basel, Switzerland). Lapatinib (HY-50898) were purchased from MedChemExpress (Princeton, NJ, USA). Primary antibodies used for immunohistochemistry (IHC) assays were the following: Rabbit mAb against p-Akt (Ser473) (Cat. #4060, 1:100 dilution), rabbit mAb against p-HER3 (Y1289) (Cat. #4791, 1:100 dilution), and rabbit mAb against HER3 (cat# 12708, 1: 400 dilution) from Cell Signaling Technology (Beverly, MA, USA); Rabbit polyclonal Ab against p-FOXO3a (Ser253) (Cat. #PA5-36816, 1:50 dilution) and mouse mAb against IRS1 (Cat. #MA5-36222, 1:50 dilution) from Thermo Fisher Scientific Inc. (Waltham, MA, USA).

### Cells and cell culture

Human BC cell lines SKBR3 and BT474 were obtained from the American Type Culture Collection (Manassas, VA). The trastuzumab-resistant sublines SKBR3-pool2 and BT474-HR20, derived from SKBR3 and BT474, respectively, were described previously [26, 27]. They were routinely maintained in the presence of 20μg/ml of trastuzumab. Cell line authentication was confirmed by Short Tandem Repeat (STR) analysis with PowerPlex® 18D System from Promega (Madison, WI, USA). All cell lines were free of mycoplasma contamination, determined by the MycoAlert™ Mycoplasma Detection Kit (Lonza Group Ltd. Basel, Switzerland) every six months. Cells were cultured with DMEM/F-12 (1:1) medium (Thermo Fisher Scientific) containing 10% fetal bovine serum (FBS) (Thermo Fisher Scientific) in a 37°C humidified atmosphere containing 95% air and 5% CO2 and split twice a week.

### Tumor xenograft model

Athymic nu/nu female mice were purchased from Charles River Laboratories Inc. (Wilmington, MA) and maintained according to the procedures and guidelines approved by the Institutional Animal Care and Use Committee (IACUC). SKBR3 or SKBR3-pool2 cells were suspended in 100μL of PBS, mixed (1:1) with Matrigel (BD Biosciences, Franklin Lakes, NJ) and inoculated subcutaneously into the right flank of female athymic mice to generate xenograft tumors. Tumor formation was measured with fine calipers twice a week. Tumor volume was calculated by the formula: Volume = (Length × Width^2^)/2, where length was the longest axis and width the measurement at a right angle to the length. When SKBR3-pool2 tumor volume reached ~ 80mm^3^, the tumor-bearing mice received intraperitoneal (i.p.) injections of PBS or trastuzumab (20 mg/kg). Tumor growth curves were plotted using average tumor volume and followed by statistical analysis as described previously [29, 32]. To examine the antitumor activity of lapatinib against trastuzumab-resistant BC, SKBR3-pool2 or BT474-HR20 cells were suspended in 100μL of PBS, mixed with Matrigel (BD Biosciences), and inoculated subcutaneously into the right flank of female athymic mice to generate xenograft tumors. When tumor sizes reached ~ 80 mm^3^, mice were randomly divided into 2 groups and treated with vehicle control (DMSO) or lapatinib (80mg/kg, i.p.). The treatment was administered every other day. Tumor growth was measured every 3 days. At the end of treatment, mice were sacrificed, and the tumors were dissected and measured for weight. All tumors were collected for further analysis.

### Immunohistochemistry (IHC) assay

IHC assays were performed as we described previously [47–49]. In brief, the tumors were formalin-fixed and paraffin-embedded. Five-micron-thick sections were deparaffinized in xylene and rehydrated with a series of graded alcohols. The slides were then treated with 3% hydrogen peroxide in methanol for 15 min. To exhaust endogenous peroxidase activity, the antigens were retrieved in 0.01 M sodium cirate buffer (pH 6.0) using a microwave oven. The slides were blocked with a blocking sniper (Biocare Medical, Pacheco, CA), and then incubated with a primary Ab described in the figure legends at 4°C overnight. After washing with Tris Buffer Saline (pH 8.0), the slides were incubated with a MACH 1 HRP Polymer detection kit (Biocare Medical) according to the manufacturer’s instructions. The staining colors were developed with a DAB Chromogen Kit (Biocare Medical). Finally, all sections were counterstained in Mayer’s hematoxylin, nuclei blued in 1% ammonium hydroxide (v/v), dehydrated, and then mounted with permanent aqueous mounting medium (Bio-Rad). Two individuals independently evaluated the IHC slides.

### Quantification of IHC assays

Quantification of IHC assays was conducted as we reported using ImageJ and ImageJ plugin IHC profiler [47]. Briefly, the IHC images were imported into the software and followed by color deconvolution with IHC profiler. The images were then inverted into 8-bit grayscale type under the “Edit” menu of ImageJ. The “Measure” function of ImageJ was used to examine the mean intensity of the IHC images. Three fields of each IHC slide were evaluated and followed by statistical analysis.

### Statistical analysis

Experimental data were statistically analyzed using two-sided student’s t tests. Significance was set at the *P* < 0.05. All values are reported at the mean ± SD.

## Data Availability

All data generated for this study are included within this article. The data used and analyzed in the current study are available from the corresponding authors on reasonable request.

## Abbreviations

BC: Breast cancer
RTK: Receptor tyrosine kinase
TKI: Tyrosine kinase ihibitor
EGFR: Epidermal growth factor receptor
HER2: Human epidermal growth factor receptor-2
HER3: Human epidermal growth factor receptor-3
mAb: Monoclonal antibody
IRS1: Insulin receptor substrate-1
IGF-I: Insulin-like growth factor-I
IGF-1R: IGF-I receptor
PTEN: Phosphatase and tensin homolog
PI-3 K: Phosphoinositide 3-kinase
MAPK: Mitogen-activated protein kinase
IHC: Immunohistochemistry
